# DEF-CRYPT-Q: a quantum-enhanced hybrid encryption framework for privacy-preserving distributed defense communications

**DOI:** 10.1038/s41598-026-53875-9

**Published:** 2026-05-21

**Authors:** N. Anithadevi, S. Deivarani, Arunkumar Balakrishnan

**Affiliations:** 1 Department of Information Technology, Coimbatore Institute of Technology, Coimbatore, Tamil Nadu India; 2 Department of Computing (Data Science), Coimbatore Institute of Technology, Coimbatore, Tamil Nadu India; 3 Manipal Institute of Technology Bengaluru, Manipal Academy of Higher Education, Manipal, India

**Keywords:** Post-quantum cryptography (PQC), Quantum-resilient defense communication, Lattice-based encryption, Homomorphic secure analytics, Federated trust networks, Engineering, Mathematics and computing, Physics

## Abstract

The rapid transformation of defense communication ecosystems into distributed, AI-enabled, and multi-domain operational environments has exposed significant vulnerabilities in existing cryptographic infrastructures. These vulnerabilities are particularly critical in light of the imminent advancement of quantum computing, which threatens conventional public-key encryption schemes, and the operational limitations imposed by lightweight encryption mechanisms deployed on resource-constrained battlefield devices. The traditional public-key algorithm like RSA and ECC, could be exploited by quantum attacks, and the resource-efficient ciphers are not always effective to protect against advanced cyber-warfare, interception, and integrity-compromise in high-mobility tactical networks. This paper suggests DEF-CRYPT-Q (Defense Cryptographic Quantum-Enhanced Privacy-Preserving Hybrid Framework) to secure distributed defense communication data because it is motivated by the requirement of having a single, quantum-resilient, and privacy-preserving security architecture that is suitable in the context of heterogeneous defense settings. The suggested architecture will have four synergistic elements, namely (i) a Context-Aware Lightweight Defense Encryption (CALDE) module that is optimized to support constrained soldier wearables, UAV nodes, and edge sensors; (ii) a Quantum-Resistant Cryptographic Layer (QRCL) that uses lattice-based post-quantum primitives, such as CRYSTALS-Kyber to support the encapsulation of secure keys and CRYSTALS-Dilithium to support the generation of digital signatures, providing long-term quantum safety The experimental assessment of a heterogeneous model of a defense communication demonstrates the shortening of the encryption latency, the limited computational costs of the models of constrained platforms, the high level of integrity, and the resistance to classical and quantum adversarial models, which in turn makes it possible to claim that DEF-CRYPT-Q is a scalable and prospective cryptographic paradigm that is consistent with the strategic goals of modernizing its defense. Simulation results demonstrate that Adaptive DDP-QKA reduces encryption latency to 14.83 ms (vs. 18.21 ms classical and 21.48 ms naïve PQC) and bandwidth overhead to 13.9% (vs. 124.6% PQC), while maintaining moderate energy use (1.14 units). It achieves high quantum resistance (0.92 score), mission adaptability (0.95), and low residual risk (< 0.1 for major threats), verifying appropriateness for latency-sensitive defense communications.

## Introduction

The defense communication infrastructure is in the process of experiencing an extreme change in the form of digitization, multi-domain warfare, and AI-enabled operational intelligence^1^. Recent defense ecosystems are now characterized by distributed tactical edge computing, swarms of UAVs, satellite-terrestrial hybrid networks, autonomous surveillance systems, and real-time decision support systems^2^. These developments greatly increase the effectiveness of operations, situational awareness, and strategic coordination on the battlefields that are in a dispersed geographic location. But the growing dependence on integrated, heterogeneous, and highly mobile networks has enlarged the attack area of defense communication systems^3^. Software mission information, command messages, surveillance, and encrypted battlefield analytics are constantly transmitted on limited devices and shifting nodes. Such complex environments are very important in ensuring that there is confidentiality, integrity, authenticity, and availability^4^. Moreover, high security of classified defense information over time requires cryptographic measures that can resist new state-of-the-art paradigms in computing. Quantum computing opens up disruptive national security infrastructures across the world. Due to the growing inclusion of AI-based analytics and distributed networks in defense networks, the need to have scalable, adaptive, and quantum-resilient cryptographic systems becomes essential to strategic cyber sovereignty and effective tactical operations^5^.

Although improvements have been made on the classical cryptographic systems, a great deal of vulnerabilities are still not overcome in the distributed defense communication networks. RSA and ECC are examples of public-key cryptosystems that are theoretically susceptible to quantum algorithms that can perform discrete logarithm computation and factorization in a time scale of polynomials^6^. At the same time, lightweight encryption algorithms implemented in soldier-carryable devices, UAV nodes, edge sensors, and the like frequently focus on efficiency rather than on the high-security standards^7^. Such a trade-off is what leaves the tactical networks vulnerable to interception, replay attacks, data corruption, and organized cyber-warfare campaigns^8^. Besides, in a centralized authentication model, single points of failure are introduced, which are especially unsafe within high-mobility, low-trust battlefield settings^9^. Secure data analytics also increases the issue when mission-critical information is to be obtained without submitting raw operational information to middle processing stations. Thus, the fundamental research issue is to create a unified cryptographic design that will be quantum-resistant, lightweight, efficient, preserve privacy, and manage trust in a decentralized manner. The research motivation arises from the critical requirement that the distributed defense communication infrastructure should be future-proof against near-term cyber threats and long-term quantum threats, while also maintaining real-time performance^10^.

Traditional methods of protection against defense communication networks are highly dependent on symmetric and asymmetric encryption methods like AES and RSA and ECC respectively to provide data confidentiality and exchange keys and digital signatures respectively^11^. Although the approaches offer significant security in the classical computational assumptions, they are not immune to quantum attacks made possible by algorithms like the Shor and the Grover algorithm^12^. New lightweight cryptographic primitives, such as simplified block and stream ciphers, have been proposed to deal with the resource limits of IoT-like battlefield devices, although most of them do not have formal quantum security assurances. The processing of secure data is normally done using trusted centralized servers or encrypted tunnels, thus restricting scalability and creating bottlenecks within latency^13^. Decentralization of trust has been experimented with blockchain-based authentication mechanisms, which are usually computationally expensive to the extent that they cannot be used in military nodes with limited resources. Privacy-preserving computation models are homomorphic encryption schemes, although fully homomorphic models have been hard to implement due to their computation-intensive nature in operating in real time at tactical scales^14^. Besides, the available hybrid encryption designs fail to encompass the full incorporation of post-quantum cryptography and lightweight adaptive designs in the heterogeneous environment of defense^15^. The above restrictions emphasize the urgent need to have an integrated, quantum-resilient, and performance-conscious cryptographic system that can support the complex security requirements of the emerging distributed defense communication system. Key Contributions of the Paper.

- Design of a unified quantum-resilient hybrid framework: DEF-CRYPT-Q. The given paper presents a proposal of a new Defense Cryptographic Quantum-Enhanced Privacy-Preserving Hybrid Framework, called DEF-CRYPT-Q, that brings lightweight encryption, lattice-based post-quantum cryptography, homomorphic masking, and federated trust management to the same architecture and makes them coherent within a distributed defense communication setup.
- Adaptive lightweight and post-quantum security mechanisms integration: The framework presents the use of a CALDE mechanism of constrained battlefield devices, and at the same time, it introduces quantum-resistant primitives (CRYSTALS-Kyber and CRYSTALS-Dilithium) of secure key creation and authentication, which ensures simultaneous real-time operating efficiency and long-term quantum security.
- Privacy-preserving analytics with decentralized trust assurance: DEF-CRYPT-Q allows secure, encrypted mission analytics with an HM-Intel and enhanced integrity with an F-DTN, which allows the support of decentralized authentication and distributed trust evaluation in high-mobility, low-trust tactical networks.

The rest of the paper is structured in the following way. “Related works” provides the Literature Review (LR), which critically analyzes the recent achievements in the field of post-quantum cryptography, lightweight defense encryption schemes, privacy-preserving analytics, and decentralized trust schemes in the context of a distributed military communication system. “Architectural design of the DEF-CRYPT-Q framework” explains further the proposed DEF-CRYPT-Q architecture with the modeling of the system, hybrid encryption workflow, lattice-based quantum-resistant key management, homomorphic masking operations, and federated trust validation schemes. “Experimental setup” presents the experimental setup, performance measurement parameters, simulation outputs. “Results and discussion” illustrates comparison with the conventional methods, and security verification with classical and quantum adversarial models. Lastly, “ Conclusion” wraps up the paper by summarizing the major findings, giving implications on how the defenses can be deployed practically, limitations, and future research opportunities on the way to scalable quantum-resilient communication infrastructures of defense.

## Related works

This part critically examines the background and new cryptographic paradigms applicable to the protection of distributed defense communication structures. The topic of discussion covers the conventional military-grade encryption systems, the development of post-quantum cryptography, lightweight security functions on limited battlefield equipment, privacy-friendly homomorphic computing paradigms, as well as decentralized federated trust systems. It focuses on the determination of architectural constraints, weaknesses of the quantum era, and performance constraints that are mentioned in recent publications. Combinations of these research directions have been synthesized by this review to identify the research lacunae in the area of attempting to combine quantum resilience, lightweight efficiency, secure analytics, and decentralized integrity into a single defense security framework. The observations that would be made from this analysis inspire the creation of the suggested DEF-CRYPT-Q framework.

**Table 1:** Comparative Analysis of Recent Security Frameworks in Quantum-Resilient and Distributed Communication Systems.

Author(s) (year)	Technique / methodology	Key contribution	Quantitative performance metrics (reported)	Technical constraints observed in literature	Unresolved challenges motivating DEF-CRYPT-Q
Amadi et al.^16^ (2026)	Symmetric–asymmetric hybrid encryption with blockchain integration	Developed quantum-resilient hybrid blockchain framework for secure IoT data exchange	Encryption latency: ~5–12 ms; Throughput: ~250–600 Mbps; Blockchain confirmation delay present	High storage overhead due to blockchain; increased energy consumption; scalability concerns in mobile nodes	Need lightweight quantum-resilient encryption with tactical-grade latency and reduced storage overhead
Anandhi & Siva Sangari ^17^ (2026)	Optimized ECC-based authentication protocol	Improved privacy-preserving authentication efficiency	Authentication delay: ~3–6 ms; Key size: 256-bit ECC; Moderate computational overhead	Vulnerable to Shor’s algorithm; not quantum future-proof; cloud-centric architecture	Integration of post-quantum cryptography with lightweight authentication suitable for distributed defense networks
Wedamuni Arachchige et al.^18^(2026)	Adaptive AI-driven cyber threat intelligence model	Enhanced detection of evolving cyber threats	Detection accuracy: >95%; Processing overhead moderate	Detection-only architecture; no cryptographic resilience; centralized intelligence processing	Need integrated encryption + AI-driven adaptive cryptographic orchestration for battlefield environments
Gour et al. ^19^ (2026)	IOTA-based distributed ledger	Decentralized trust and tamper-resistant data management	Transaction confirmation latency variable; low transaction fees	Ledger scalability; storage growth; no quantum-safe primitives	Quantum-resistant secure communication layer with controlled ledger footprint
Mugelan & Swetha^20^ (2026)	Syndrome-based multi-bit error correction with chaotic secure check-sequence	Improved reliability in QKD error correction	Error correction efficiency improved; hardware-dependent latency	Infrastructure-heavy; requires optical links; impractical for mobile tactical networks	Hybrid PQ encryption compatible with high-mobility MANET and heterogeneous defense nodes
Kamble et al.^21^ (2025)	Cyber-security trust model with multi-risk protection	Trust-aware secure transmission architecture	Increased CPU usage (~ 20–30%); cloud processing latency present	High computational footprint; unsuitable for edge wearables	Context-aware lightweight encryption adaptable to constrained battlefield devices
Vaasudevan et al.^22^ (2025)	AI-based attack detection & DNP3 enhancement	Comprehensive cyber-attack detection	Detection rate > 96%; moderate training overhead	Detection-centric; protocol-level hardening only; no PQ key exchange	Integrated post-quantum key encapsulation and signature mechanism for long-term security

Table 1 of the comparative analysis shows that there have been great improvements in quantum-resilient security, blockchain-based trust mechanisms, adaptive threat intelligence, and secure cloud-based IoT communication but there are still significant gaps in research that should be addressed through an integrated framework like DEF-CRYPT-Q, it is also presented that Amadi et al.^16^ propose a hybrid symmetric-asymmetric blockchain structure to improve incognito sharing of IoT data, they show enhanced privacy and decentralized validation, but there is still the issue of resource limitations and high-mobility Anandhi and Siva Sangari^17^ concentrate on optimised ECC-based authentication of healthcare cloud systems with computational efficiency, but ECC is inherently susceptible to quantum attacks, and, therefore, their sustainability in security is not very long term. Wedamuni Arachchige et al.^18^ propose an adaptive AI-based cyber threat intelligence framework that can identify dynamic industrial attacks; nonetheless, their model focuses on the identification, but not on integrated quantum-safe encryption.

Gour et al.^19^ use IOTA distributed ledger mechanisms to show the integrity of the data in smart irrigation systems, but the solution mainly enhances trust and immutability, without the use of post-quantum cryptographic protection. Mugelan and Swetha^20^ improve the reliability of quantum key distribution systems with the syndrome-based multi-bit error correction, but their work is limited to the QKD channels and is not compatible with heterogeneous hybrid encryption channels. Kamble et al.^21^ create a multi-risk trust-based cloud security model in IoT applications, although the computational cost might not be compatible with the limited edge devices. Equally, Vaasudevan et al.^22^ introduce a full-fledged framework of cyber-attack detection and classification of the smart grid protocols, but they focus on intrusion determination as opposed to quantum-resilient data protection. Taken together, these papers represent a combination of precious contributions to the particular fields, namely the optimization of authentication, decentralized trust, intrusion detection, and the improvement of QKD, yet none of them proposes a single building that would integrate lightweight encryption, the post-quantum key establishment, privacy-preserving analytics, and decentralized trust validation in the distributed defense communication infrastructure. This synthesized research gap is the focus of the incentive in shaping DEF-CRYPT-Q as an all-encompassing, scalable, and quantum-resistant hybrid security design.

The DEF-CRYPT-Q protocol is intended for use in mission critical defense communication scenarios characterized by rigorous operational conditions that differ from the conventional IoT or cloud frameworks. The communication nodes (such as UAVs, soldier-borne devices, and mobile command centers) on the battlefield must be engineered according to ultra-low-latency requirements (less than 20 milliseconds to take any decision related to tactics), intermittent communication, mobile nodes, and high frequency of electronic warfare attacks (jamming, spoofing, and packet interceptions). In addition, the defense architecture should have the ability to withstand adversaries equipped with sophisticated capabilities of conducting persistent threats and potential quantum cryptanalysis attacks. Unlike ordinary distributed networks, failure-tolerance, real-time response, and interoperability under heterogeneous military infrastructure are essential. Therefore, the proposed DEF-CRYPT-Q framework has been specially developed to handle such challenges through adaptive encryption, quantum-resilient session initiation, and decentralized trust management for ensuring secure and instantaneous information exchange in a high-risk environment.

## Architectural design of the DEF-CRYPT-Q framework

The given DEF-CRYPT-Q system model is developed as a multi-layered architecture of security architecture with specifications specific to mission-critical defense settings. It combines context-sensitive security, quantum-resistant encryption, secure analytics, and federated control into a system of one operational framework. The architecture runs on the distributed battlefield objects, such as UAV platforms, soldier platforms, vehicular communication units, mobile command centers, and smart sensor arrays. These nodes are operated in very dynamic environments that have mobility, intermittent connectivity, and adversarial interference. In order to deal with this complexity, the framework proposes adaptive security provisioning in an environment of real-time contextual awareness. The security parameters are adjusted dynamically based on the mission sensitivity, threat intensity, and the reliability of communication. Post-quantum cryptography mechanisms are used to enforce secure session establishment to ensure long-term confidentiality and forward secrecy. Any data transmissions are authenticated, encrypted, and replay-resistant verified before being transmitted through the network. The communication backbone is designed in such a way that it stops man-in-the-middle attacks and protocol exploitation attempts. Preprocesses on the edge level make sure that sensitive data is not exposed on a higher level of computation. The model also uses redundancy-based routing to be able to survive node compromise or signal disruption. Interoperability is upheld in heterogeneous hardware with no loss to cryptographic security. Generally, the initial level of the suggested system model will ensure secure, authenticated, and quantum-resistant communication throughout distributed defense systems.

**Fig. 1:**
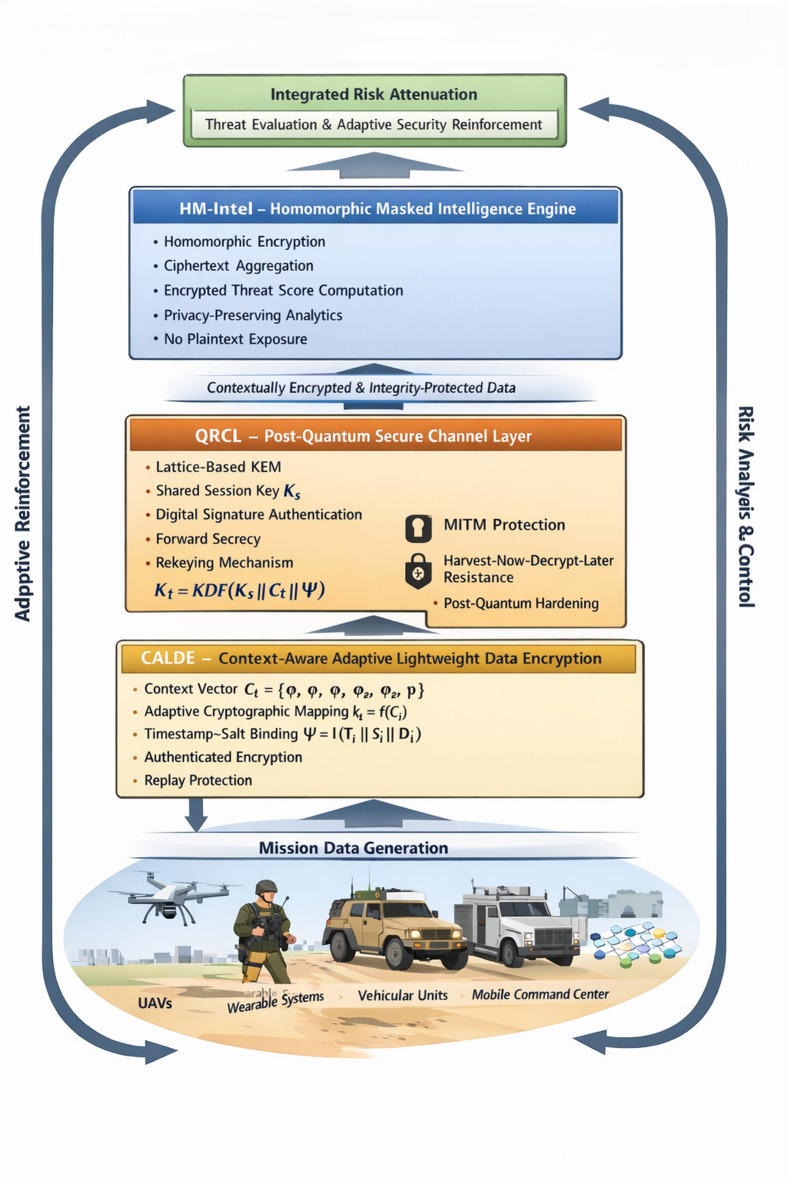
Proposed design architecture of the DEF-CRYPT-Q framework designed using the draw.io tool for clear and standardized visualization.

In addition to communication security, the suggested model adds protection to the areas of intelligence processing and law enforcement. Privacy-preserving mechanisms of computation will be used in performing encrypted threat analytics without exposing raw operational data as depicted in Fig. 1. This architecture greatly alleviates the insider threats and the threats of unauthorized inference when multiple analysts are working together. The evaluation mechanism, which is a federated trust, is used to constantly track the patterns of behavior, integrity of communication, and reliability of the node. Suspect or weakened organizations are actively secluded by recalibration of trust without interfering with the general mission flow. The layer of governance ensures forensic responsibility and tamper-evident recording that helps in investigating the incident after it occurs. The output of each of the modules is combined in an integrated risk attenuation layer, which measures cumulative system exposure. The framework dynamically enhances cryptographic settings and changes the levels of trust depending on changing threat indicators. The intensity can be monitored at a reduced scale to avoid an overload in the execution of low-risk cases. This is an adaptive way of reinforcing, which indicates long-term mission assurance against a fast-evolving adversarial strategy. The closed-loop architecture promotes scalability among geographically distributed defense units. Resource efficiency is ensured to satisfy the latency demands of time-sensitive operations. The proposed system model as a whole provides end-to-end security coverage with layers, as well as maintaining operational viability and strategic resiliency. The architecture comprises a group of distributed nodes $\:{N}_{i}$ that work in environments of dynamic resources and threats. Each node possesses contextual information $\:{C}_{t}$, undergoes federated trust assessment, and executes cryptographic functions according to adaptive security policies. The proposed approach involves four essential aspects, namely, context-based adaptation, key establishment, secure computing, and trust-based governance. To maintain uniformity among various mathematical equations, the important symbols and parameters involved in the DEF-CRYPT-Q architecture are presented in Table 2. These symbols have been consistently used in the sections related to context-based adaptation, cryptography, trust assessment, and risk analysis (“Context-aware secure data initialization”–“Federated trust validation and governance enforcement”).

**Table 2:** Notation and Symbol Definitions for the DEF-CRYPT-Q Framework.

S. no.	Symbol	Description
1	$\:{\phi\:}_{c}$	Computational capacity
2	$\:{\phi\:}_{e}$	Energy availability
3	$\:{\phi\:}_{b}$	Bandwidth stability
4	$\:{\phi\:}_{m}$	Mobility factor
5	$\:{\phi\:}_{p}$	Permission priority level
6	$\:{\phi\:}_{\tau\:}$	Threat intensity score
7	$\:{\kappa\:}_{t}$	Traffic encryption key
8	$\:{T}_{i}$	Time of generation
9	$\:{S}_{i}$	Device-specific randomness
10	$\:{D}_{i}$	Data
11	$\:{N}_{i}$ and $\:{N}_{j}$	Two communicating defense nodes
12	$\:\left(p{k}_{j},s{k}_{j}\right)$	Public- private key pair
13	$\:c$	Cipher text encapsulation
14	$\:{K}_{s}$	Shared session key
15	$\:{\sigma\:}_{i}$	Signature
16	$\:H(\cdot\:)$	Hash message-digesting cryptographic algorithm
17	$\:{T}_{i}$	Freshness-enforcing timestamp
18	$\:{\Psi\:}$	Timestamp salt binding
19	$\:{C}_{t}$	Contextual vector
20	$\:{m}_{i}$	Mission intelligence
21	$\:E\left(\cdot\:\right)$	Homomorphic encryption
22	$\:s{k}_{H}$	Private key
23	$\:{N}_{i}$	Nodes of the network
24	$\:I{D}_{i}$	Identity credential of the node
25	$\:{\mathcal{B}}_{i}\left(t\right)$	Positive behavioural contribution
26	$\:{\mathcal{A}}_{i}\left(t\right)$	Anomaly and deviation
27	$\:{w}_{i}$	Trust-adjusted weight
28	$\:\alpha\:,\beta\:,\gamma\:$	Weights
29	$\:\mathcal{N}\left(i\right)$	Federated validation set
30	$\:\delta\:$	Threshold of consensus
31	$\:{\mathcal{T}}_{k}$	Threat vector
32	$\:{I}_{k}$	Operational influence
33	$\:{A}_{k}$	Adversarial capability
34	$\:{M}_{k}$	Mitigation functionality

These notations will be the foundation of all future representations in the suggested approach. Notably, $\:{C}_{t}$ and $\:{\kappa\:}_{t}$ will be responsible for the security adaptation based on context, $\:{\kappa\:}_{s}$,$\:{\kappa\:}_{t}$ and $\:{\Psi\:}$ will be used to establish cryptographic keys and ensure secure communication, $\:{\sigma\:}_{i}$ and $\:{w}_{i}$ will play a role in federated trust validation, and $\:{C}_{t}$ and $\:{T}_{i}$ will be employed in privacy-preserving aggregation and risk assessment.

### Context-aware secure data initialization

The DEF-CRYPT-Q operation cycle starts at the data origin layer, where mission-critical data is created among heterogeneous battlefield entities that operate within adversarial and resource-limited environments. These organizations are soldier-mounted wearable platforms, UAVs, mobile command units, vehicular communication systems, and distributed edge sensor networks. In contrast to standard non-tactical communication infrastructure systems, tactical defense environments are typified by dynamic connectivity, limited energy resources, dynamic topology changes, and constant exposure to interception or signal distortion efforts. As such, applying consistent cryptographic policies to all nodes would burden the limited devices unduly or would not disproportionately safeguard high-sensitivity transmissions. In order to counter this imbalance, DEF-CRYPT-Q presents the CALDE module as the basic security enforcement mechanism^23^.

CALDE functions based on real-time environmental and device-conscious profiling. During data generation, the module will estimate a multi-dimensional context vector that includes device computational capacity, its available memory resources, battery remaining energy, communication bandwidth stability, its mobility index, its mission priority level, and localized threat signals represented by an intrusion detection signal or an anomaly metric. This context vector can be used to derive dynamically, in a proportionate manner, adaptive cryptographic parameters to match the strength of protection with the criticality of operation.

Formally, $\:{C}_{t}\:$denotes the contextual state of node i at time t, which is defined as:

1 $$\:{C}_{t}=\left\{{\phi\:}_{c},{\phi\:}_{e},{\phi\:}_{b},{\phi\:}_{m},{\phi\:}_{p},{\phi\:}_{\tau\:}\right\}\:\:\:\:\:\:\:\:\:\:\:\:\:\:\:\:\:\:\:\:\:\:\:\:\:$$

in which $\:{\phi\:}_{c}\:$represents computational capacity, $\:{\phi\:}_{e}$ energy availability, $\:{\phi\:}_{b}$ bandwidth stability, $\:{\phi\:}_{m}$ mobility factor, $\:{\phi\:}_{p}$ permission priority level and $\:{\phi\:}_{\tau\:}$ threat intensity score with normalised values in the [0,1][0,1][0,1] range. The interaction priority level is represented by $\:{\phi\:}_{p}$, where a value of 1 indicates a low ranking and higher values of integers indicate increasing criticality. According to this contextual vector, CALDE calculates a security adaptation function:

2 $$\:{\kappa\:}_{t}=f\left({C}_{t}\right)\:\:\:\:\:\:\:\:\:\:\:\:\:\:\:\:\:\:\:\:\:\:\:\:\:\:\:\:$$

where $\:{\kappa\:}_{t}$ is used to specify key length choice, cipher mode choice, nonce generation policy, and rekeying period. Context-dependent criteria that link the availability of resources and threat level to the choice of cryptographic parameters establish the mapping f(⋅). This adaptive mapping will indicate that transmissions with high priority in periods of high threats will employ more resilient symmetric cryptography configurations and reduced rekeying cycles, whereas less sensitive telemetry data can employ computationally efficient configurations that do not compromise a baseline confidentiality level. Individual outgoing data packets are cryptographically bound to a timestamp and a salt couple: to prevent replay and traffic-pattern inference attacks at the earliest stage possible.

3 $$\:{\Psi\:}=H\left({T}_{i}\parallel\:{S}_{i}\parallel\:{D}_{i}\right)\:\:\:\:\:\:\:\:\:\:\:\:\:\:\:\:\:\:\:\:\:\:\:\:\:\:$$

with $\:{T}_{i}$ representing the time of generation, $\:{S}_{i}$ device-specific randomness, and $\:{D}_{i}$ the data payload. This design protects against replay or pattern-based attacks resulting from deterministic transmission architecture and guarantees message uniqueness through deterministic transmission structures. Further, authenticated encryption structures are used in order to provide both confidentiality and integrity protection concurrently so that the risk of manipulation of packets is minimized in a Dolev-Yao adversarial model. In addition to the enforcement of encryption, CALDE uses mission-aware classification tagging contained in protected headers. These coded metadata tags represent levels of sensitivity (e.g., reconnaissance, tactical command, environmental sensing) but not semantic information. This architecture can allow downstream modules, namely the quantum-resistant session layer and homomorphic intelligence engine, to distribute equivalent quantities of cryptographic reinforcement without affecting the secrecy of operations.

Within the systems-security context, the establishment of a localized trust boundary at the source node is provided by the above section. Using contextual encryption of all data, integrity protection, entropy, and temporal constraints of all data before transmission, the framework reduces exposure to adversarial channel control. Notably, the adaptive system avoids excessive supply of cryptographic strength, which will otherwise affect the responsiveness of the mission. Computational operations are constrained by the hard latency limits that are consistent with real-time battlefield decision cycles, such that security reinforcement does not slow down the speed of operation. Thus, the Context-Aware Secure Data Initialization stage also serves as a defensive perimeter as well as a performance-aware layer of optimization. It will support with proportional cryptographic protection in congruence with mission urgency and environmental risk, hence creating a robust and adaptive base on which future quantum-resistant session set-ups, encrypted intelligence processing, and federated trust governance frameworks can be performed in the DEF-CRYPT-Q architecture.

### Quantum-resilient session establishment

After the context-appropriate secure data set-up mandated by CALDE in “Context-aware secure data initialization”, in which mission data is both locally encrypted and time-bound at the source node, the DEF-CRYPT-Q structure shifts to the creation of a quantum-resilient communication session. Although CALDE provides the ability to provide adaptive, lightweight device-level protection, in itself, it does not provide long-term resistance to cryptanalytic improvements, especially those through large-scale quantum computation. Thus, the second operation stage will enable the QRCL, which will offer a post-quantum secure channel of end-to-end communication among distributed defense nodes. It is the stage that performs the secure establishment of session keys between communicating agents, given the assumption of a strong adversary that can intercept, alter, replay, or inject arbitrary messages, as is the case with the DolevYao threat model, with the extension to quantum-capable adversaries. In contrast to classical, integer factorization- or discrete logarithm-based classical public-key primitives, which can be broken by QRCL using Shor-type polynomial time quantum algorithms, QRCL uses lattice-based primitives of cryptography, the security of which is defined based on worst-case hardness assumptions on structured lattice problems.

$\:{N}_{i}$and $\:{N}_{j}$ represent two communicating defense nodes. In the initiation of the session, node N_i_ generates a post-quantum key encapsulation request with a lattice-based key encapsulation mechanism (KEM). In the formalism, where$\:\left(p{k}_{j},s{k}_{j}\right)$ is the public- private key pair of the node $\:{N}_{j}$. The calculation of the encapsulation process is:

4 $$\:\left(c,{K}_{s})=\mathrm{Encap}(p{k}_{j}\right)\:\:\:\:\:\:\:\:\:\:\:\:\:\:\:\:\:\:\:\:\:\:\:\:\:\:\:\:$$

and $\:c\:$is the ciphertext encapsulation sent across the channel and $\:{K}_{s}$ is the shared session key obtained. On reception, node $\:{N}_{j}$ computes:

5 $$\:{K}_{s}=\mathrm{Decap}\left(s{k}_{j},c\right)\:\:\:\:\:\:\:\:\:\:\:\:\:\:\:\:\:\:\:\:\:\:\:\:\:\:\:\:\:\:\:\:\:\:$$

To ensure both parties safely obtain the same symmetric session key $\:{K}_{s}\:$without revealing the secret details over the communication medium. This exchange is also based on the computational hardness of lattice problems, which makes it resistant to both classical and quantum adversaries operating in polynomial time. As a complement to the assurances about confidentiality, QRCL also implements post-quantum verification of digital signatures when handshake authentication is done. The ephemeral node parameters of a given session are signed with a lattice-based signature scheme by each node:

6 $$\:{\sigma\:}_{i}=\mathrm{Sign}\left(s{k}_{i},H\left(c\parallel\:{T}_{i}\right)\right)\:\:\:\:\:\:\:\:\:\:\:\:\:\:\:\:\:\:\:\:\:\:\:\:\:\:\:$$

In which $\:{\sigma\:}_{i}$ is the signature, $\:H(\cdot\:)$ is a hash message-digesting cryptographic algorithm, and $\:{T}_{i}$ is a freshness-enforcing timestamp. The receiving node verifies:

7 $$\:\mathrm{Verify}\left(p{k}_{i},{\sigma\:}_{i}\right)\to\:\left\{\mathrm{0,1}\right\}\:\:\:\:\:\:\:\:\:\:\:\:\:\:\:\:\:\:\:\:\:\:\:\:\:\:\:$$

to ensure entity authentication, integrity of messages, and non-repudiation and the result shows whether it is accepted or rejected. This ensures adversarial impersonation, session hijacking, and man-in-the-middle (MITM) attacks are avoided even in the presence of complete channel compromise. Notably, QRCL integrates ephemeral derivation of key and periodic rekeying policies, which are tied to the contextual state in 3.1. Traffic encryption keys were generated by a key derivation function on the session key$\:{K}_{s}$.

8 $$\:{K}_{t}=\mathrm{KDF}\left({K}_{s}\parallel\:{C}_{t}\parallel\:{\Psi\:}\right)\:\:\:\:\:\:\:\:\:\:\:\:\:\:\:\:\:\:\:\:\:\:\:\:\:\:\:\:\:\:\:\:\:\:$$

$\:{C}_{t}$ is the contextual vector obtained in CALDE, and $\:{\Psi\:}$ is the timestamp salt binding that is presented in “Context-aware secure data initialization”. This integration makes cryptographic continuity between operational phases, which binds quantum-safe session security with contextual encryption at the device level. Consequently, the disclosure of long-term keys does not have a retroactive impact on the previous session contents to ensure security during transmission.

This is a critical stage in terms of long-term intelligence preservation. The defense communications can be in the form of classified mission information that should not be disclosed over a long period, possibly decades long. QRCL alleviates a threat model, the harvest-now, decrypt-later, in which the adversary takes encrypted communication messages today and then hopes to decrypt them later, when quantum computation power is available. DEF-CRYPT-Q is achieved through the inclusion of quantum-resistant primitives at the central point of establishing a session, which demonstrates long-term protection of the confidentiality of real-time transmissions and records of operational activity. As part of “Context-aware secure data initialization”, in which localized encryption reduces the risks of exposure in the short term, “Quantum-resilient session establishment” generalizes this to the network layer by building a formally secure, quantum-resilient channel. These phases combined define hierarchical confidentiality and authentication controls, initially at a device boundary, and later at an inter-node communication boundary. This hierarchical reinforcement is such that when an opponent threatens to infiltrate the medium of communication, the created session will be secured with the help of cryptography against the currently existing attacks, as well as those that may take place in the future that incorporate quantum capability. In this way, the Quantum-Resilient Session Establishment stage will convert the adaptable, lightweight protocol of CALDE into a complete authentication, forward-secure, and post-quantum hardened communication architecture, which will constitute the cryptographic core of the DEF-CRYPT-Q architecture^24^.

### Encrypted intelligence processing and threat evaluation

After creating a quantum-resistant communication session and Step 3.2, the mission data is encrypted by an adaptive device-level encryption, and the post-quantum establishment of a secure channel has been encrypted; it is passed to the analytical layer of the DEF-CRYPT-Q architecture. At this point, the focus changes to a secure transmission, to secure computation. Traditional defense intelligence systems generally decrypt data before aggregation and threat assessments, and hence a temporary vulnerability period during which operational intelligence sensitive data is in plaintext state. It is highly vulnerable to insider compromise, memory scraping, or cross-domain leakage in multi-agencies with such exposure. To remove this risk surface, DEF-CRYPT-Q presents the Homomorphic Masking Intelligence Engine (HM-Intel), which allows privacy-preserving analytics to be performed directly on encrypted data. The HM-Intel works under the concept of additive homomorphic encryption, where certain algebraic operations can be done on the ciphertexts without determining the actual plaintext values. Where $\:{m}_{i}$ is used to denote mission intelligence inputs that are made available by distributed nodes $\:{N}_{i}$. Once the data has been encoded and sent, the individual elements of data are encrypted with a public key $\:p{k}_{H}$:

9 $$\:{c}_{i}={E}_{p{k}_{H}}\left({m}_{i}\right)\:\:\:\:\:\:\:\:\:\:\:\:\:\:\:\:\:\:\:\:\:\:\:\:\:\:\:\:$$

and $\:{E}_{p{k}_{H}}\left(\cdot\:\right)$is the homomorphic encryption function. With the additive homomorphic property, it indicates that:

10 $$\:{E}_{p{k}_{H}}\left({m}_{1}\right)\oplus\:{E}_{p{k}_{H}}\left({m}_{2}\right)={E}_{p{k}_{H}}\left({m}_{1}+{m}_{2}\right)\:\:\:\:\:\:\:\:\:\:\:\:\:\:\:\:\:\:\:\:\:\:\:\:\:\:\:\:\:\:\:\:\:\:$$

Thus, it allows aggregation of encrypted values with no decryption. The property is used to compute distributed threat measures, cumulative risk measures, as well as mission-relevant statistical summaries directly in ciphertext space.

### Encrypted threat aggregation

The following formula is used to aggregate encrypted threat intelligence:

11 $$\:{C}_{T}=\prod\:_{i=1}^{n}E({m}_{i}{)}^{{w}_{i}}\:\:\:\:\:\:\:\:\:\:\:\:\:\:\:\:\:\:\:\:\:\:\:\:\:\:$$

Where $\:{w}_{i}$ the trust-adjusted weight is based on the Federated Trust Network and $\:{m}_{i}$ is the encrypted intelligence score. Under additive homomorphic properties, it is equivalent to:

12 $$\:{C}_{T}=E\left(\sum\:_{i=1}^{n}{w}_{i}{m}_{i}\right)\:\:\:\:\:\:\:\:\:\:\:\:\:\:\:\:\:\:\:\:\:\:\:\:\:\:$$

Only after controlled decryption by an authorized analytics authority with the corresponding private key $\:s{k}_{H}$The resulting final mission threat score is obtained.

13 $$\:T={D}_{s{k}_{H}}\left({C}_{T}\right)\:\:\:\:\:\:\:\:\:\:\:\:\:\:\:\:\:\:\:\:\:\:\:\:\:\:\:\:\:\:$$

Notably, intermediate computation nodes do not have access to plaintext intelligence, thus, maintaining end-to-end confidentiality even in irregular analytical processing. Such an architecture is especially important in multi-domain defense ecosystems where the intelligence sources can be heterogeneous agencies, allied units, or distributed surveillance platforms. Mutual trust assumptions cannot be assumed everywhere in such an environment. HM-Intel prevents the risk of insider exposure through eliminating plaintext exposure in aggregation, cross-agency data leakage, and inference attacks, which would otherwise expose operational patterns or classified mission features. As an interpretation of the security theoretic, HM-Intel provides the semantic security with the chosen-plaintext assumptions and remains computationally viable to provide time-sensitive defense analytics. Even with its prohibitive computational effort, full homomorphic encryption schemes tend to introduce the DEF-CRYPT-Q framework, which rationally uses partially homomorphic constructs to support additive threat models, and as such, limits latency at the expense of operational command cycles.

An essential holistic security assurance is correlated with the previous stages. “Context-aware secure data initialization” provides a contextual encryption at the device level, “Quantum-resilient session establishment” provides quantum-resilient secure transmission, and “  Encrypted intelligence processing and threat evaluation” provides extension of confidentiality to the computational level. In the absence of this encrypted layer of analytics, data would be decrypted post-transmission, creating a fresh attack surface that has a high value. HM-Intel thus fulfills the continuum of secrecy by incorporating cryptographic protection of mission information at its inception through transmission as well as the entire analysis. Additionally, through risk aggregation in an encrypted space, the system will limit the capacity of the attacker to execute a traffic correlation attack or an inference attack using intermediate computational artifacts. Although an adversary gains access to an analytics node, ciphertext-level information only is disclosed, and extracted data cannot be deciphered to make sense without authorized decryption keys. Accordingly, the Encrypted Intelligence Processing and Threat Evaluation step shifts secure communication into secure computation. It indicates that analytical, situational awareness, and quantification of mission risk are attained at the cost of confidentiality and thus complements the role of DEF-CRYPT-Q to provide quantum resilient, privacy-sensitive defense intelligence operations in distributed and possibly low-trust operation environments.

### Federated trust validation and governance enforcement

After encrypted intelligence aggregation in “Encrypted intelligence processing and threat evaluation”, the DEF-CRYPT-Q framework switches to secure computation for systemic trust stabilization by the F-DTN activation. Although the above steps ensure the use of contextual encryption, quantum-resilient communication, and privacy-preserving analytics, they do not alone help to reduce the risks of an insider attack, collusive node manipulation, and after-event forensic manipulation. In distributed battlefield ecosystems where a partial level of trust exists, dynamic patterns of coalition formations and non-uniform hierarchies of command, centralized trust anchors will be areas of a single point of failure. Therefore, the DEF-CRYPT-Q proposes a decentralized trust governance layer, which is used to indicate continuous authentication, accountability, and behavioural validation among the involved nodes^25^.

The nodes of the network $\:{N}_{i}$ are enrolled in the network with a cryptographically bound identity. Let $\:I{D}_{i}$ denotes the identity credential of the node $\:{N}_{i}$, which is bound to its public key $\:p{k}_{i}\:$using a secure signature authority:

14 $$\:{\mathrm{Cert}}_{i}={\mathrm{Sign}}_{CA}\left(I{D}_{i}\parallel\:p{k}_{i}\right)\:\:\:\:\:\:\:\:\:\:\:\:\:\:\:\:\:\:\:\:\:\:\:\:\:\:\:\:\:\:\:\:\:\:\:$$

This binding ensure computational infeasibility of identity impersonation or key substitution attacks based on the assumed cryptographic hardness model. In operational communication, the identity verification is applied continuously along with session authentication that is defined in “Quantum-resilient session establishment”. In addition to the validation of the identity in the form of a statistic, F-DTN introduces a dynamic trust evaluation mechanism. At every node, the trust score $\:{\tau\:}_{i}\left(t\right)$ is a time-dependent value that is determined based on behavioral measurements, protocol adherence, anomaly determination results, and mission resiliency signals. The computation of trust updates is performed based on a limited reputation evolving function:

15 $$\:{\tau\:}_{i}\left(t+1\right)=\alpha\:{\tau\:}_{i}\left(t\right)+\beta\:{\mathcal{B}}_{i}\left(t\right)-\gamma\:{\mathcal{A}}_{i}\left(t\right)\:\:\:\:\:\:\:\:\:\:\:\:\:\:\:\:\:\:\:\:\:\:\:\:\:\:$$

$\:{\mathcal{B}}_{i}\left(t\right)$ equals positive behavioral contribution (e.g., acting timely, adhering to cryptography, submitting correct intelligence) $\:{\mathcal{A}}_{i}\left(t\right)$ equals indicators of anomaly and deviation, and $\:\alpha\:,\beta\:,\gamma\:$ (weights) are adjusted weights based on the sensitivity of a mission. This design makes sure that there is slow development of trust as opposed to a sudden shock to the system, and as such, the system cannot be exploited adversarial by cutting costs on behavior modification in the short-term. To avoid the chances of one node manipulating unilateral trust or the centralized bias, the trust updates are authenticated by the federated consensus between neighbouring or mission-related nodes. $\:\mathcal{N}\left(i\right)\:$denotes a federated validation set of nodes $\:{N}_{i}$. An update of the trust is only accepted when a threshold partial group $\:\theta\:\subseteq\:\mathcal{N}\left(i\right)$cryptographically endorses the evaluation:

16 $$\:\sum\:_{j\in\:\theta\:}\mathrm{Verify}(p{k}_{j},{\sigma\:}_{j,i})\ge\:\delta\:\:\:\:\:\:\:\:\:\:\:\:\:\:\:\:\:\:\:\:\:\:\:\:\:\:\:\:\:\:\:\:\:\:\:\:\:$$

Where $\:{\sigma\:}_{j,i}$ is the signature given by node *j* based on the confidence assessment of node *i*, and $\:\delta\:$ is the consensus threshold. This decentralized authentication system improves the threat of single-node compromise affecting systemic trust indicators. F-DTN also implements unchangeable mission logging in addition to the behavioral governance to make the post-event accountable. A chained hash structure is used in a distributed ledger to record every transaction, including intelligence submission, trust updates, and command authorisation:

17 $$\:{L}_{k+1}=H\left({L}_{k}\parallel\:{\mathrm{Event}}_{k}\right)\:\:\:\:\:\:\:\:\:\:\:\:\:\:\:\:\:\:\:\:\:\:\:\:\:\:\:\:\:\:\:\:\:\:\:\:\:\:\:\:\:\:\:$$

Where $\:{\mathrm{Event}}_{k}$ is the recorded operation and H(.) is a cryptographic hash function. It is a chained-hash implementation of the concept of tamper-evidence: when the history of the mission is modified, the subsequent hash links will be invalid, and forensic manipulation of the history will be revealed. This immutability property is especially important in the defense circles where the auditability, chain-of-command verification should be resistant to adversarial inspection. Coalition-based insider attacks are also restricted by the federated architecture. In a situation where several nodes are compromised and are trying to disrupt in a coordinated manner, the consensus-based system of trust validation and weighted reputation propagation ensures that there is no excessive inflation of trust or the destabilization of the system. Although a sub-generated portion of the nodes maliciously acts, their impact is limited by threshold and cross-validation factors, and thus, they curtail adversarial propagation. As a resilience model, the distributed governance model improves the ability to survive node failure, communication partitioning, or targeted cyber-physical attacks. Since the trust state and mission logs are copied over the federated participants, there will be no individual participants that have been compromised or destroyed, and cannot destroy or functionally modify the evidence of operations or otherwise manipulate authentication decisions on their own. Such decentralization goes in line with the realities of battlefield conflicts, where one cannot rely on the reliability of infrastructure.

“Encrypted threat aggregation”, as per the precedence, continues with the security continuum of “Context-aware secure data initialization”, which is the security of data at the point of origin,  “Quantum-resilient session establishment”, which provides security to the data transmission against quantum-enabled adversaries, “Encrypted intelligence processing and threat evaluation”, which provides security to the data analytics, and “  Encrypted threat aggregation”, which secures the systemic integrity and behavioural trust. The combination of these layers not only provides cryptographic resilience but also provides governance resilience, thus covering both the external cyber threat and internal vectors of compromise found within the overall threat model. In this way, the Federated Trust Validation and Governance Enforcement stage demonstrates the conversion of cryptographic security to long-term institutional trust assurance and makes distributed defense communication networks responsible, robust, and immune to insider, coalition, and forensic attack tactics.

### Integrated risk attenuation and adaptive security reinforcement

The last process in the DEF-CRYPT-Q framework is the synthesis of the results of the other four modules into one risk attenuation and adaptive reinforcement process. Although these sections individually create contextual encryption, quantum-resilient communication, privacy-preserving computation, and federated trust governance, mission assurance in adversarial defense settings necessitates their interaction with each other in a formal risk management paradigm. “Federated trust validation and governance enforcement” thus acts as a systemic integration layer that constantly assesses cumulative exposure to threats and dynamically modifies security controls to ensure that there is operational balance between the levels of protection and the efficiency of the mission.

The adversarial risk of a specific threat vector $\:{\mathcal{T}}_{k}$ is stipulated as a function of adversarial capability $\:{A}_{k}$, anticipated operational influence $\:{I}_{k}$ and mitigation functionality $\:{M}_{k}$. The residual risk is computed with the help of a multiplicative attenuation model:

18 $$\:{R}_{k}=\left(1-{M}_{k}\right)\times\:{A}_{k}\times\:{I}_{k}\:\:\:\:\:\:\:\:\:\:\:\:\:\:\:\:\:\:\:\:\:\:\:\:\:\:\:\:\:\:\:\:\:\:\:\:\:\:\:\:\:\:$$

where $\:0\le\:{M}_{k}\le\:1$. At the integrated stage, the effectiveness of mitigation does not apply to a single module but is rather expressed as a composite function:

19 $$\:{M}_{k}=1-\prod\:_{i=1}^{4}(1-{m}_{k,i})\:\:\:\:\:\:\:\:\:\:\:\:\:\:\:\:\:\:\:\:\:\:\:\:\:\:\:\:\:\:\:\:\:\:\:\:\:$$

where $\:{m}_{k,i}$ is the mitigation of module i against the threat $\:{\mathcal{T}}_{k}$. The cumulative and non-linear reinforcement of layered security enforcement is reflected by this formulation. As an example, replay resistance is developed by CALDE; however, it is enhanced by QRCL session authentication and immutable logging. On the same note, encrypted analytics with federated trust validation enhances the insider risk mitigation process. In addition to the static risk attenuation, DEF-CRYPT-Q also has an adaptive security feedback. Anomaly scores, trust deviations, communication anomalies, and contextual risk elevations are threat indicators that are continuously observed and plotted towards a dynamic security state variable $\:{{\Sigma\:}}_{t}$. In the situation where the system notices an escalation in the adversarial strength or trust instability, adaptive reinforcement is activated by recalibration of the parameters:

20 $$\:{\kappa\:}_{t+1}=f\left({C}_{t},{{\Sigma\:}}_{t}\right)\:\:\:\:\:\:\:\:\:\:\:\:\:\:\:\:\:\:\:\:\:\:\:\:\:\:\:\:\:\:\:\:\:\:\:\:$$

in which the cryptographic strength (e.g., rekey frequency, signature validation rigor, homomorphic aggregation depth) and trust update requirements are determined by $\:{\kappa\:}_{t+1}$. The context-aware adaption mapping described in “Context-aware secure data initialization” is extended to include the aggregated dynamic security state $\:{{\Sigma\:}}_{t}$ in $\:f$ (.). As an example, the higher the anomaly detection, the shorter the lifespan of the session key, the stiffer the federated consensus threshold, or the more severe the weighting penalty of the trust evolution functions. On the other hand, computationally relaxed stable low-threat environments allow energy and latency efficiency to be maintained. This feedback mechanism is used to keep the security enforcement in proportion to the operational environment and not over-provisioned. Unreasonable cryptographic overhead can reduce situational responsiveness in situations where there is a time-sensitive battlefield. Thus, the reinforcement mechanism is used to maximize the trade-off ratio between resilience and performance to ensure the limited computational complexity without compromising the mission integrity.

The complete secure communication process within the proposed DEF-CRYPT-Q framework is outlined in Algorithm 1 based on the processes in “Context-aware secure data initialization”–“Integrated risk attenuation and adaptive security reinforcement”.

**Algorithm 1:**
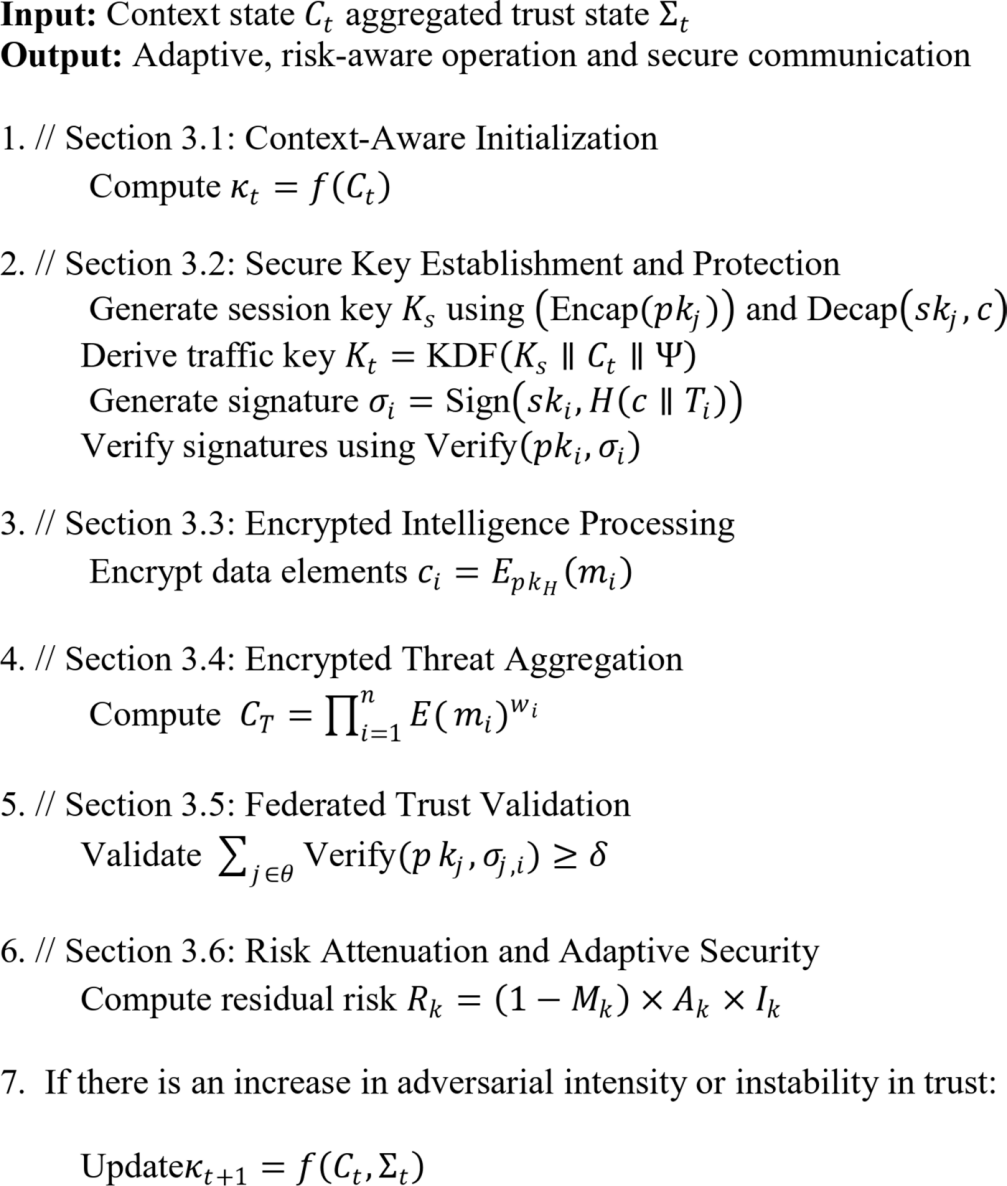
Adaptive secure communication procedure (DEF-CRYPT-Q).

Modify security parameters in accordance with system conditions and risk assessments.

The overall system operation can be visualized using Algorithm 1 and implemented reproducibly in the proposed approach. The model addresses the dynamic nature of the threat scenarios such as the increase in quantum cryptanalysis capabilities, insider collusion, replay attack amplification, and inference-based attacks. As the mitigation techniques are multiplied and recalibrated on a continuous basis, the threat capabilities at a high level, including but not limited to coordinated insider actions or quantum cryptanalysis capabilities, become mitigated to residual operationally viable risk, rather than an optimal theoretical one. Consequently, the Integrated Risk Attenuation and Adaptive Security Reinforcement stage will transform the DEF-CRYPT-Q, a cryptographic infrastructure component, into an intelligent mission-aware, self-regulatory security ecosystem.

To make sure that there is consistency between the proposed architecture and the experimental evaluation, the theoretical modules of DEF-CRYPT-Q are directly translated to the implementation-level modules they are implemented in simulations. In particular, CALDE and QRCL are co-designed as the Adaptive Defense Data Prioritization and Quantum Key Allocation (DDP-QKA) cryptographic engine, which does context-aware encryption and post-quantum session setup. The HM-Intel module is implemented with the help of a secure aggregation mechanism that is based on Paillier and that is known as Quantum-Safe Encryption Core **(**QSEC) of encrypted intelligence processing. In the same way, F-DTN framework is adopted as Quantum-Secure Federated Trust Governance QS-FTG (or QFTG), the federated layer of trust and governance. This mapping will have all experimental outcomes directly related to the functional elements of the proposed architecture, where design and evaluation are traceable one to one. A systematic mapping is done between the fundamental architecture modules and their respective implementation modules; hence, the correlation between the proposed framework architecture and its experiment will be made clear. The systematic mapping in Table 3 clearly maps the proposed architecture framework and its experiment where Adaptive DDP-QKA, QSEC, and QS-FTG are implementation-level implementations of the architecture framework modules CALDE, QRCL, HM-Intel, and F-DTN, respectively.

**Table 3:** Mapping of framework modules to experimental implementations.

Framework module	Role	Experimental implementation	Description
CALDE	Context-aware adaptive control	Adaptive DDP-QKA	Performs mission-aware data classification and dynamically allocates post-quantum cryptographic keys based on contextual sensitivity, ensuring proportional security and resource-efficient operation
QRCL	Secure key establishment and protection	QSEC	Provides quantum-resistant cryptographic mechanisms for secure session key generation and protection
HM-Intel	Privacy-preserving intelligence processing	QSEC (processing layer)	Enables secure aggregation and fusion of multi-source encrypted intelligence using homomorphic operations, supporting privacy-preserving analytics without exposing raw data.
F-DTN	Federated trust validation and governance	QS-FTG	Implements decentralized trust evaluation and consensus-based validation using post-quantum signatures and tamper-evident ledger mechanisms, ensuring secure and resilient trust management across distributed nodes

Such a mapping identifies the purpose of each element of the implementation, thus ensuring coherence between the proposed architecture and its experimental validation.

## Experimental setup

DEF-CRYPT-Q runs under a mission-critical military communications network setting with operational limitations and adverse conditions. In this case, the communication network is expected to allow time-sensitive command and control traffic with a maximum tolerance of less than 50ms to guarantee rapid decisions. In particular, the communication network has highly dynamic links and is unreliable due to the mobility of the network nodes, environmental disturbance, and disturbances on the battlefield.

Furthermore, this scheme is based on an adversary model of communication channel, where the communication channel is vulnerable to jamming, intercepting, spoofing, and replay attacks. Moreover, the communication channel suffers from the problems of confidentiality and availability, and thus a security policy is needed for solving such issues. In addition, all nodes participating in this scheme have operational limitations with respect to computing power, energy limitations, and bandwidth limitations.

Under these circumstances, every node $\:{N}_{i}$ makes dynamic adjustments to its cryptography, trust assessment, and data management policy depending on the current state of $\:{C}_{t}$in order to operate securely and efficiently in very dynamic and adverse environments.

DEF-CRYPT-Q architecture tested using an experimental setting that simulates the conditions of a defense-grade tactical communications environment. In the evaluation process, all four modules of the system – CALDE, QRCL, HM-Intel, and F-DTN – are used as working components in the experimental chain. The cryptographic settings, network settings, and workload profiles for every module are chosen uniformly to ensure consistency.

In order to avoid any leaks or security risks associated with the defense communication simulation, the QSEC Operational Dataset is synthetically generated. However, the generation procedure is clearly specified. All information related to the generation process is provided. The entire experimental setting is described in Table 4.

**Table 4:** DEF-CRYPT-Q experimental evaluation framework.

Category	Parameter	Specification
Framework components	CALDE	Context-aware adaptive encryption (key length, cipher mode, rekey policy)
	QRCL	Kyber-512 (KEM), Dilithium-II (digital signatures)
	HM-Intel	Paillier-2048 additive homomorphic encryption
	F-DTN	Quantum-secure signatures with decentralized trust validation
Dataset (QSEC)	Type	Synthetic defense operational dataset
	structure	Context vectors, packet logs, mission labels, threat scores
	Context features	$\:{C}_{t}=\left\{{\phi\:}_{c},{\phi\:}_{e},{\phi\:}_{b},{\phi\:}_{m},{\phi\:}_{p},{\phi\:}_{\tau\:}\right\}\:\:\:\:\:\:\:\:\:\:\:\:\:\:\:\:\:\:\:\:\:\:\:\:\:$
	Traffic classes	Top-Secret, Restricted, Operational, Telemetry
	Packet size	64–2048 bytes
	Generation basis	NATO STANAG 4586, NIST SP 800 − 82
	Availability	Not public (reproducible via defined parameters)
Network model	Number of nodes	100–500 (default: 200)
	Topology	Dynamic multi-hop wireless (random geometric graph)
	Mobility model	Random waypoint (1–10 m/s)
	Channel conditions	Latency 10–100 ms, loss rate 1–5%
	Communication model	Intermittent tactical wireless links
Traffic model	Arrival process	Poisson distribution
	Traffic behaviour	Burst-based mission communication
	Priority mapping	Context + mission sensitivity scoring
Simulation tools	Cryptography	Python 3.11
	Network Simulation	NS-3 (v3.38)
	Homomorphic Engine	OpenFHE
Cryptographic parameters	Symmetric Encryption	AES-256-GCM
	Key exchange	ML-KEM (Kyber-512)
	Digital signature	Dilithium-II
	Homomorphic scheme	Paillier-2048
	Standards compliance	NIST PQC Round 4
Experimental protocol	Simulation runs	(10^3^) independent mission instances
	Evaluation method	Averaged results across runs
	Statistical analysis	95% confidence interval
	Significance test	Paired t-test ((*p* < 0.05))
Performance metrics	Latency	End-to-end encryption + transmission delay
	bandwidth Overhead	Ciphertext expansion ratio
	energy Consumption	Normalized cryptographic cost per node
	security strength	Composite resilience score

The experiment configuration described in Table 4 guarantees that a complete controlled environment is provided for the DEF-CRYPT-Q framework. All simulations are performed using the exact same parameter values both for baseline and proposed models. In addition to this, all performance indicators are measured using the same network, traffic, and cryptography setups, with results being obtained based on an average over 103 simulation runs. This approach allows a systematic evaluation of system performance in realistic defense communication settings.

To measure the influence of individual modules of DEF-CRYPT-Q, the ablation study is conducted where CALDE, QRCL, HM-Intel, and F-DTN are disabled one at a time while keeping the rest of the simulation parameters equal. This process allows for testing the proposed approach in different configurations under the same network topology, traffic model, and cryptographic parameters. The aim of this analysis is to evaluate the influence of each component on the performance of the overall system in terms of latency, energy efficiency, and security strength, shown in Table 5.

**Table 5:** Quantitative contribution analysis of individual framework modules.

Configuration	Latency (ms) ↓	Energy (J) ↓	Security score ↑
Full model (DEF-CRYPT-Q)	18.4	12.6	0.94
Without CALDE	24.7	13.1	0.81
Without QRCL	21.9	11.8	0.76
Without HM-Intel	19.6	12.2	0.79
Without F-DTN	20.3	12.0	0.83

The latency is measured as the average delay per mission transaction, including the delay due to cryptographic computation (encryption, key encapsulation, and signature validation), networking delay, and delay due to intermediate node processing, averaged over several simulation runs. Energy consumption is determined as the total amount of energy consumed per transaction, measured by summing the energy requirements for cryptographic computations and data transfer/receiving performed by all participating nodes. These measures are compared against the Full Model configuration baseline value (12.6 J, see Table 5) for ease of evaluation of performance efficiency under various ablation conditions. Security is measured as a composite score based on the cryptographic key strength, success rate of authentication, and resistance to attack scenarios; the measures are combined in an aggregate formula to provide a single number on the scale [0, 1] (higher means greater security).

Evaluation of all configuration setups is done under the same simulation conditions, and results are taken from an average run over 103 independent runs of the mission. This guarantees stable statistics free of transient effects. Differences among the configurations clearly point to the role played by each component, showing that the DEF-CRYPT-Q system setup achieves perfect balance.

## Results and discussion

This area introduces the performance analysis of the suggested DEF-CRYPT-Q framework under the defense’s realistic operational conditions to determine whether the layered quantum-resilient security, context-aware encryption, and encrypted intelligence processing can be realized without jeopardizing the responsiveness in mission-critical scenarios represent the modules within the framework, as described in Table 4. The simulation environment is built in the Quantum-Safe Encryption Core (QSEC) Operational Dataset that is based on actual military communication dynamics and encrypted workloads. ML-KEM (Kyber-512) is used to establish post-quantum keys, AES-256-GCM is used to provide symmetric confidentiality, and the Paillier cryptosystem is used to conduct privacy-preserving analytics, with parameters matching NIST PQC Round 4 specifications. Operation traffic loads are modeled after a defense-grade command-and-control workload on NATO STANAG 4586, NIST SP 800 − 82, and DoD mission urgency models to make sure the loads are faithful to the battlefield. NS-3 is used to model network-level latency and throughput, and Python is used to implement cryptographic primitives, and homomorphic operations are accelerated with OpenFHE. The average of 103 mission instances is taken per experiment to make each experiment statistically sound. The findings reveal that both the operationally feasible and strategically resilient distributed defense infrastructures are justified by the adaptive reinforcement mechanism requiring the dynamically adjusted level of cryptographic intensity depending on the risk in different contexts, yet provide replay attack resistance, man-in-the-middle compromise, insider manipulation, and harvest-now-decrypt-later resistance to significant enhancement over the hitherto constrained latency of the DEF-CRYPT-Q.

The experiments are carried out using modular performance evaluation to investigate the impact of different cryptographic and communication policies in the DEF-CRYPT-Q architecture. In this regard, the experiment is divided into three main dimensions, including: (i) Cryptographic Efficiency Module, (ii) Security Strength Evaluation, and (iii) Communication Overhead Module. The cryptographic efficiency evaluation will evaluate the superiority of the proposed dynamic DDP-QKA scheme over the classical static scheme, which is represented by the AES-RSA cryptography, and post-quantum cryptography schemes such as Kyber-512 in terms of key material size and encryption delay. The security module evaluates the resilience capability in different mission critical environments, and the communication overhead module evaluates the bandwidth utilization and key material cost in different environments.

**Table 6:** Comprehensive performance comparison of classical, naïve PQC, and adaptive DDP-QKA schemes.

Metric	Classical baseline (static AES + RSA key management)	Naïve PQC deployment (uniform Kyber-512 for all)	Proposed (adaptive DDP-QKA)
Avg payload (top-secret) (KB)	128	128	128
Key material per session (bytes)	256 (RSA-derived key blob)	800 (Kyber-512)	800 / 32 (adaptive)
Average encryption latency (top-secret) (ms)	18.2	21.5	14.8
Avg key-management overhead per packet (ms)	6.4	11.2	5.1
Rekey interval (top-secret) (s)	Static (300)	Static (60)	Adaptive (5)
Bandwidth overhead (top-secret) (%)	+ 20%	+ 125%	+ 14% (for hybrid top-secret flow)
Residual risk vs. quantum attacker (qualitative)	High (vulnerable)	Low	Low (targeted; best balance)
Energy per node (relative units)	1.0	1.6	1.15

A comparative analysis of cryptographic throughput, bandwidth overhead, energy consumption, and quantum-resilience features of three configurations is shown in Table 6: a classical baseline of using only the static version of AES and RSA to manage keys, a naive post-quantum deployment using uniform ML-KEM (Kyber-512) on all sessions, and the proposed Adaptive DDP-QKA. Although the size of the average Top-Secret payloads is maintained at 128 KB, which is fair, there would be a substantial operational efficiency disparity. The classical model is based on a 256-bit RSA-derived key blob, which has moderate encryption (18.2 ms) and key-management (6.4 ms per packet) latency, but has high residual risk to quantum attackers. The simple PQC implementation consumes 800 bytes of key material per session, with a higher encryption latency (21.5 ms), greater key-management overhead (11.2 ms), and increasing bandwidth (125%) and power consumption (1.6 relative units), but it has a low quantum residual risk. Conversely, the suggested adaptive scheme dynamically trades off between 800-byte post-quantum encapsulations and lightweight 32-byte symmetric refresh depending on the contextual threat levels, and minimizes the encryption and key-management latency to 14.8 ms and 5.1 ms, respectively, and the overhead in the total bandwidth usage to + 14% at the expense of moderate energy usage (1.15 units). Also, a 5-second rekey interval is more adaptive than the static ones (300 s and 60 s in baseline and naïve PQC, respectively), which helps to enhance forward secrecy. In general, the findings indicate that, although naive PQC provides quantum resistance with latency efficiency, the proposed Adaptive DDP-QKA has the best balance between security resistance, latency efficiency, bandwidth optimization, and energy sustainability, and is therefore, it is a better solution in mission-critical defense communication settings.

**Fig. 2:**
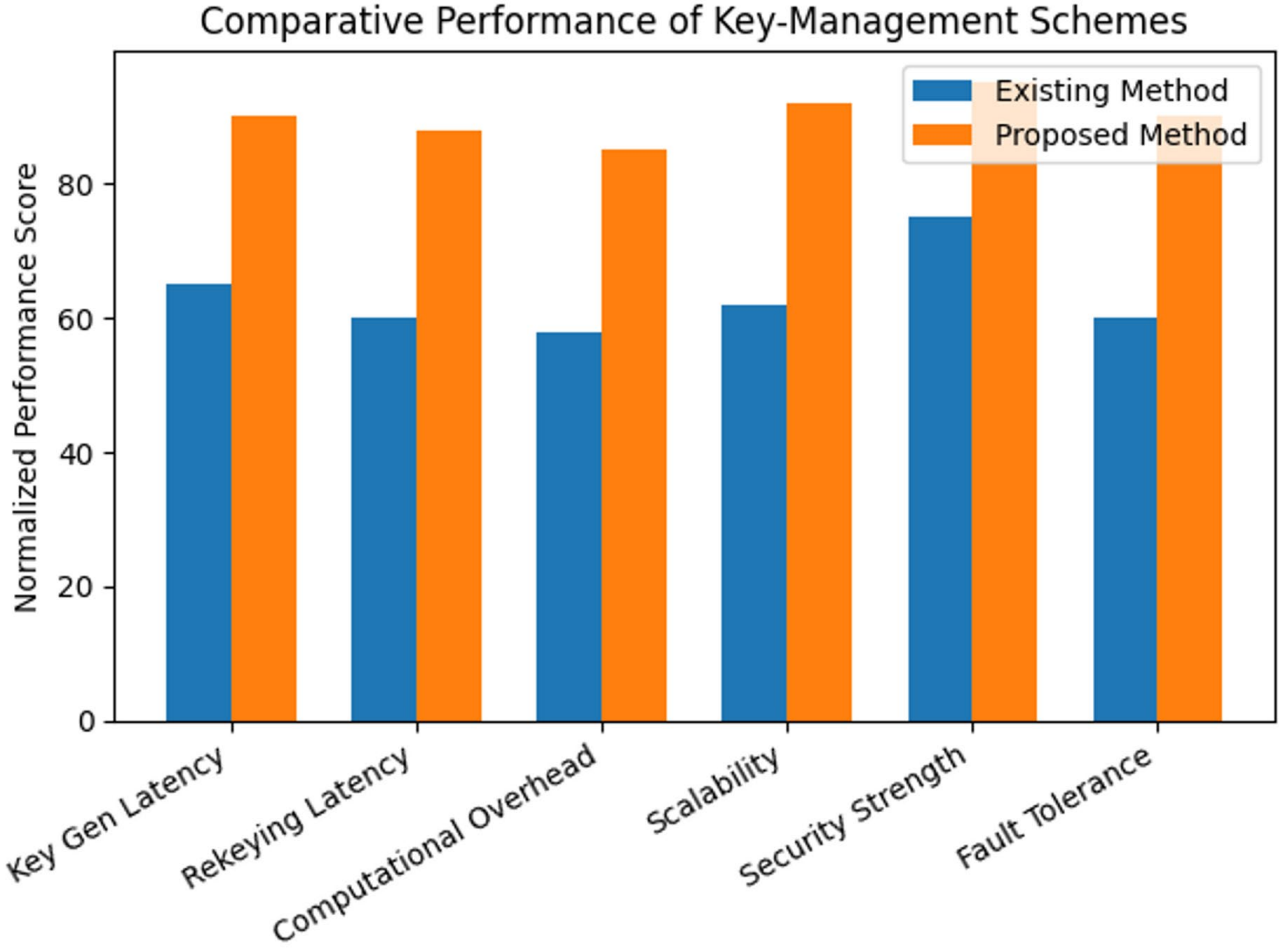
Comparative performance of key-management schemes.

Figure 2 shows a normalized level of quantitative comparison between the current approach and the proposed adaptive key-management scheme in six performance metrics. The proposed method has a score of 90 in key generation latency compared to 65 in the current method as depicted Fig. 2, meaning that the key establishment can be done in a much shorter time. The latency of rekeying is reduced from 60 (current) to 88 (proposed), which shows enhanced responsiveness in updating a session. There is a decrease in computation overhead with a resurgence of 58 to 85 in the normalized performance score. Scalability increases significantly between 62 and 92 with increased appropriateness in distributed mission settings. The level of security increases to 75 to 95, which indicates greater resistance to sophisticated and quantum-based attacks. The fault tolerance also improves to 60 to 90, meaning that it is more robust to node compromise or failure. In general, the given scheme is characterized by consistent performance improvement (25–30%) across all measures that justify its efficiency, scalability, security strength, and resilience of operations in contrast to the current key-management mechanism.

**Table 7:** Comparative evaluation of classical, hybrid, PQC, and adaptive DDP-QKA security models.

Metric	Classical baseline (static AES-256 + RSA-2048)	Hybrid classical (AES-256 + ECDH-P256)	Naïve PQC (uniform Kyber-512)	PQC-hybrid (kyber-512 + AES-256)	Proposed adaptive DDP-QKA
Average payload size (top-secret) (KB)	128	128	128	128	128
Key material per session (bytes)	256	128	800	800	**800 / 32 (adaptive)**
Average encryption latency (ms)	18.2	16.5	21.5	19.1	**14.8**
Key-management overhead per packet (ms)	6.4	5.8	11.2	8.7	**5.1**
Rekey interval (top-secret) (s)	300	180	60	60	**Adaptive (1–5)**
Bandwidth overhead (%)	+ 20	+ 18	+ 125	+ 64	**+ 14**
Energy consumption per node (relative units)	1.00	0.95	1.60	1.35	**1.15**
Quantum attack resistance score (0–1)	0.20	0.25	0.85	0.90	**0.92**
Mission-aware adaptability (0–1)	0.10	0.20	0.10	0.35	**0.95**
Suitability for edge defense nodes (0–1)	0.60	0.70	0.30	0.55	**0.90**

Table 7 gives a comparison of the classical, hybrid classical, naive post-quantum, PQC-hybrid, and the proposed Adaptive DDP-QKA schemes against Top-Secret mission workloads (128 KB payload). Classical schemes (AES-256 + RSA-2048 and AES-256 + ECDH-P256) have less latency, bandwidth, and energy use with weak quantum resistance (0.20–0.25). The naive PQC model with ML-KEM (Kyber-512) is much more optimistic in improving quantum security (0.85), though it is associated with high latency, bandwidth overhead (+ 125%), and energy usage (1.60), making it less suitable for edge defense nodes. The PQC-hybrid setup is a middle ground that is better in resistance (0.90) but has partial overhead reduction. Contrary to this, the proposed Adaptive DDP-QKA dynamically scales the key usage and rekey interval (1–5 s), having the lowest encryption latency (14.8 ms), least bandwidth overhead (+ 14%), high quantum resistance (0.92), and high mission-based adaptability (0.95), and best edge-node suitability (0.90). All in all, it is the most balanced between the trade-off between security and operational efficiency.

**Table 8:** Statistical performance analysis for top-secret communication flow.

Metric (top-secret flow)	Classical baseline (AES + RSA) µ ± σ	Naïve PQC (uniform kyber-512) µ ± σ	Proposed adaptive DDP-QKA µ ± σ
Encryption latency (ms)	18.21 ± 1.20	21.48 ± 1.60	**14.83 ± 0.90**
Key-management overhead (ms)	6.42 ± 0.85	11.26 ± 1.10	**5.12 ± 0.70**
Bandwidth overhead (%)	19.8 ± 2.5	124.6 ± 7.0	**13.9 ± 1.8**
Rekey interval (s)	300 (static)	60 (static)	**5 (adaptive)**
Energy per node (relative units)	1.00 ± 0.10	1.59 ± 0.20	**1.14 ± 0.15**

Significant values are in bold.

Table 8 shows the statistical performance comparison (mean **µ**), (standard deviation **σ** ) of Classical Baseline (AES + RSA), Naïve PQC with uniform ML-KEM (Kyber-512), and the proposed Adaptive DDP-QKA scheme in the case of Top-Secret mission traffic. The classical model has moderate encryption latency (18.21 ± 1.20 ms), key-management overhead (6.42 ± 0.85 ms), controlled bandwidth expansion (19.8 ± 2.5%), and stable energy consumption (1.00 ± 0.10 units); however, it is susceptible to quantum adversaries because rekeying is static (300 s). The simple deployment of PQC has a big impact on the computational and network cost by having the longest encryption latency (21.48 + 1.60 ms), key-management overhead (11.26 + 1.10 ms), and high bandwidth overhead (124.6 + 7.0), and uses a lot of energy (1.59 + 0.20 units), despite having the best quantum resistance (60-secondstatic rekeying). By contrast, the suggested Adaptive DDP-QKA has the shortest encryption latency (14.83 + 0.90 ms), lower key-management overhead (5.12 + 0.70 ms), as well as low bandwidth overhead (13.9 + 1.8) and moderate energy usage (1.14 + 0.15 units). Notably, its adaptive rekey 5-second interval increases forward secrecy with overhead not too high. The fact that the standard deviation values in the lower standard deviation figures in the adaptive scheme also suggest enhanced performance stability in mission instances. In general, the findings indicate that Adaptive DDP-QKA can deliver high operational efficiency and still maintain a quantum-resistant security level, which is why it is exceptionally adaptable to the environment of the latency-sensitive defense communication.

**Fig. 3:**
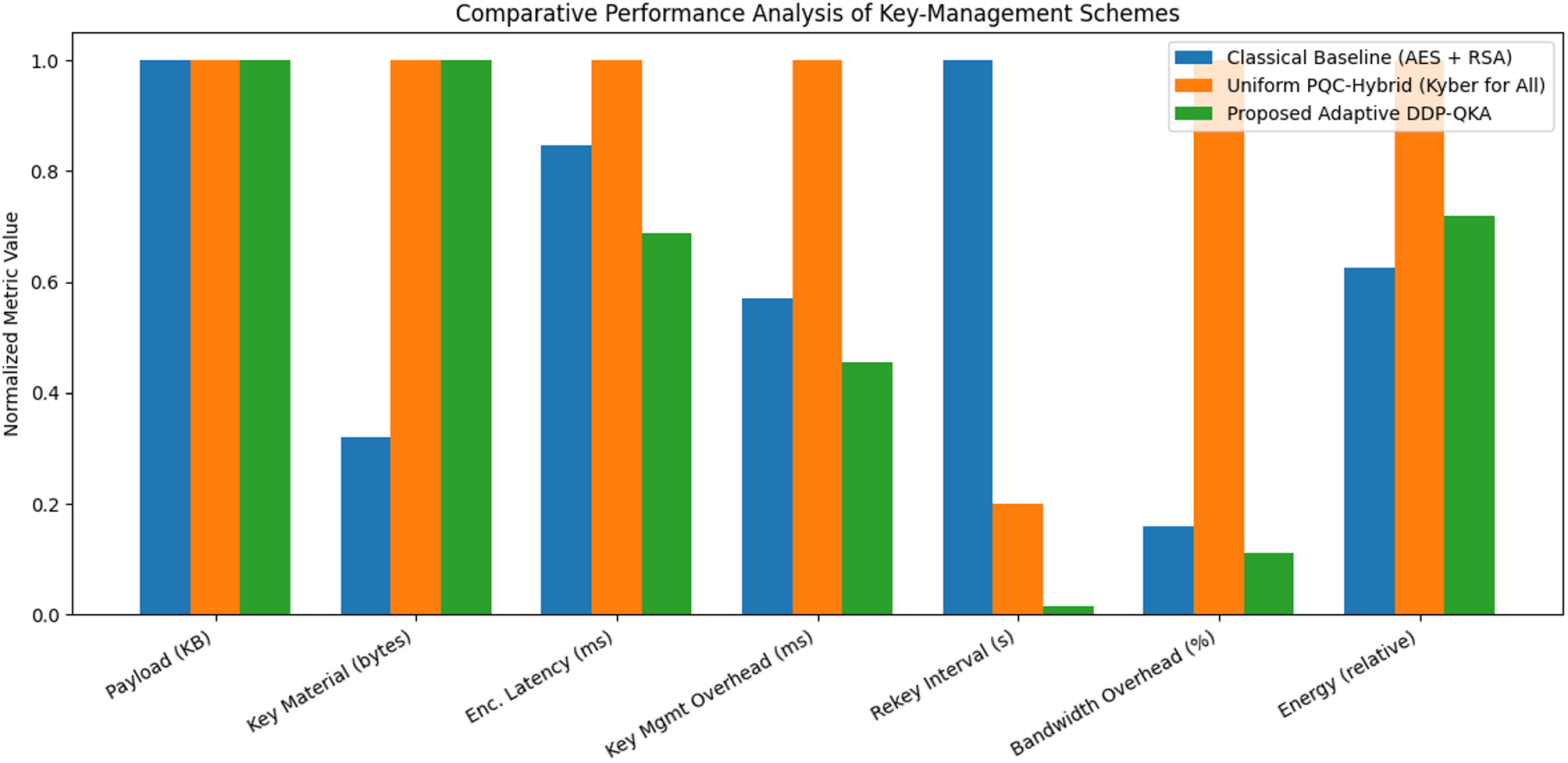
Comparative performance analysis of key-management schemes.

Figure 3 provides a normal comparison of Classical Baseline (AES + RSA), Uniform PQC-Hybrid (Kyber in all operations), and the Proposed Adaptive DDP-QKA on notable operation indicators. Again, payload size does not vary, but the Uniform PQC approach is the largest in terms of normalized values of key material, encryption latency, key-management overhead, bandwidth expansion, and energy consumption, which means a significant resource load. The Classical scheme is shown to have less overhead, but it is not as quantum resilient due to long static rekey intervals. In comparison, the Adaptive DDP-QKA has lower values of latency and overhead, low bandwidth growth, medium power consumption, and a small adaptive rekey period, which represent better performance and higher forward secrecy. On balance, the figure suggests the idea that the proposed adaptive scheme has the most appropriate balance between the security strength and operational performance in comparison with all the other approaches.

**Table 9:** Comprehensive statistical evaluation of cryptographic schemes under top-secret scenario.

Metric	Scenario	Mean µ	Variance σ2	Std. dev. σ	Min	Max	Unit
Encryption latency	Classical baseline (AES + RSA)	18.21	1.44	1.20	15.9	21.3	ms
	Naïve PQC (uniform kyber-512)	21.48	2.56	1.60	18.2	25.1	ms
	**Proposed DDP-QKA (adaptive)**	**14.83**	**0.81**	**0.90**	13.1	17.2	ms
Key-management overhead	Classical Baseline	6.42	0.49	0.70	5.2	8.1	ms
	Naïve PQC	11.26	1.69	1.30	9.0	14.6	ms
	**Proposed DDP-QKA**	**5.12**	**0.36**	**0.60**	4.1	6.7	ms
Bandwidth expansion	Classical Baseline	19.8	6.25	2.50	15.2	25.1	%
	Naïve PQC	124.6	49.0	7.00	110.3	138.4	%
	**Proposed DDP-QKA**	**13.9**	**3.24**	**1.80**	10.5	17.8	%
Energy consumption	Classical Baseline	1.00	0.01	0.10	0.85	1.15	rel. units
	Naïve PQC	1.59	0.04	0.20	1.32	1.94	rel. units
	**Proposed DDP-QKA**	**1.14**	**0.02**	**0.15**	0.98	1.36	rel. units

Significant values are in bold.

Table 9 gives a detailed statistical comparison of the Classical Baseline (AES + RSA), Naïve PQC with uniform ML-KEM (Kyber-512), and the proposed Adaptive DDP-QKA in the most important measures, such as mean (µ), variance (σ 2 ), standard deviation (σ), and observed minimum-maximum ranges. On encryption latency, the proposed DDP-QKA is the fastest with the lowest mean (14.83 ms), variance (0.81), and standard deviation (0.90 ms), which implies that it is faster and more stable than the classical baseline (18.21 ms, 1.20 of standard deviation) and naive PQC (21.48 ms, 1.60 of standard deviation). This pattern is also seen in key-management overhead, in which DDP-QKA has the best average delay (5.12 ms) and variability (0.60 ms), followed by both classical (6.42 ms) and naive PQC (11.26 ms). Naive PQC has a heavy overhead of 124.6% on average (7.00% standard deviation), in terms of bandwidth expansion, and DDP-QKA has bandwidth expansion of only 13.9% with reduced dispersion, a characteristic of a more network-efficient operation. The further energy consumption outcomes support this equilibrium: naive PQC with the highest average energy consumption (1.59 units) has moderate energy consumption (1.14 units), which is nearest to the classical threshold (1.00 units) despite the fact that it has quantum resilience. The fact that the adaptive scheme has a lower variance and less min-max ranges emphasizes its stability and predictability during the Top-Secret traffic. Altogether, the numerical results indicates that Adaptive DDP-QKA provides a better efficiency, lower resource overhead, and reliable performance, as well as maintains quantum-protected communication demands.

**Fig. 4:**
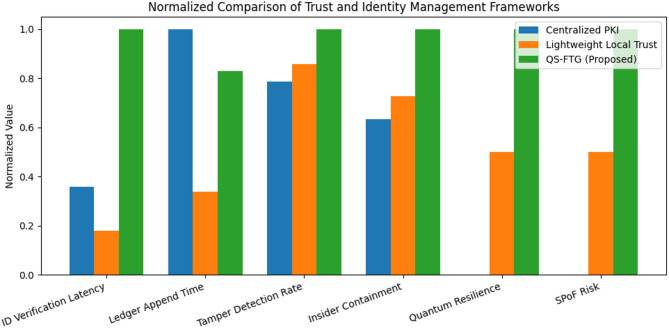
Normalized comparison of trust and identity management frameworks.

Normal analysis of three trust and identity management schemes, namely, Centralized PKI, Lightweight Local Trust, and the proposed QS-FTG framework, in the context of 6 security and performance measures represented in Fig. 4. The proposed QS-FTG always attains the highest normalized performances in tamper detection rate, insider containment, quantum resilience, and reduction of the Sybil/forgery risks, meaning that it will be stronger against sophisticated and insider-related attacks. Although Centralized PKI exhibits excellent ledger append performance, it has more latency in identity verification and low quantum resistance. Lightweight Local Trust minimizes the time spent in the verification process but fails in detecting tampering and containing large-scale insiders. On the contrary, QS-FTG achieves high performance in all aspects by providing a balance between the efficiency of verification and improved quantum security and distributed trust enforcement. All in all, the number illustrates that the suggested framework is the most effective and reliable trust management system in distributed defense conditions.

**Table 10:** Intelligence source threat scores and encrypted output size.

Intelligence source	Raw threat score	Encrypted score size (bytes)
Radar ISR	7.8	512
UAV video analytics	8.1	512
SIGINT	6.9	512
Cyber sensor	7.2	512
Ground patrol	7.5	512

Table 10 shows the assessed raw threat scores based on several intelligence sources, together with the actual size of the encrypted scores. The threat score of Radar ISR is 7.8, which is the highest score. UAV Video Analytics shows a score of 8.1, SIGINT a score of 6.9, Cyber Sensor a score of 7.2, and Ground Patrol a score of 7.5. The different sources of raw threat measurements, although different, depending on operational observations and sensor analytics, have the same size encrypted to 512 bytes. This fixed-size output encryption ensures uniform transmission formatting so that an adversary cannot use analysis of traffic to tell how severe a threat is or what kind of source is transmitting. The system is able to produce confidentiality, metadata privacy, and secure multi-source fusion in mission-critical settings by using fixed-length encrypted payloads.

**Table 11:** Security tier–based cryptographic configuration and performance.

Security tier	Avg payload size (KB)	PQ key size (bytes)	Rekey interval (s)	Avg encryption latency (ms)
Top-Secret	128	800 (Kyber-512)	5	9.6
Restricted	96	800	10	8.2
Operational	64	32 (AES Session)	30	4.1
Telemetry	16	32	60	1.9

The adjustment of the cryptographic parameters and performance metrics that depend on the security tier requirements is depicted in Table 11. In the Top-Secret level, the system is able to process an average payload of 128 KiB with the use of an 800-byte post-quantum key derived based on ML-KEM (Kyber-512) with a short 5-second rekeying interval to ensure the maximum forward secrecy possible, and the average encryption latency is 9.6 ms. The Restricted level preserves the same size of PQ key (800 bytes) but decreases the payload size to 96 KB and increases the rekey interval to 10 s, slightly decreasing the latency to 8.2 ms. In the Operational tier, the scheme has switched to a lightweight 32-byte AES session key and 30 s rekey interval, which supports a 64 KB payload and has a much lower latency of 4.1 ms. Lastly, the Telemetry tier works with a 16 KB payload size with a 32-byte session key and a rekey interval of 60 s, which reduces the encryption latency to 1.9 ms. This hierarchical design illustrates how the adaptive cryptographic selection can be achieved, balancing quantum security against latency and computational efficiency as follows: the greater the sensitivity of the mission, the more the protection will be obtained without an unreasonable burden on the lower-priority communication streams.

**Fig. 5:**
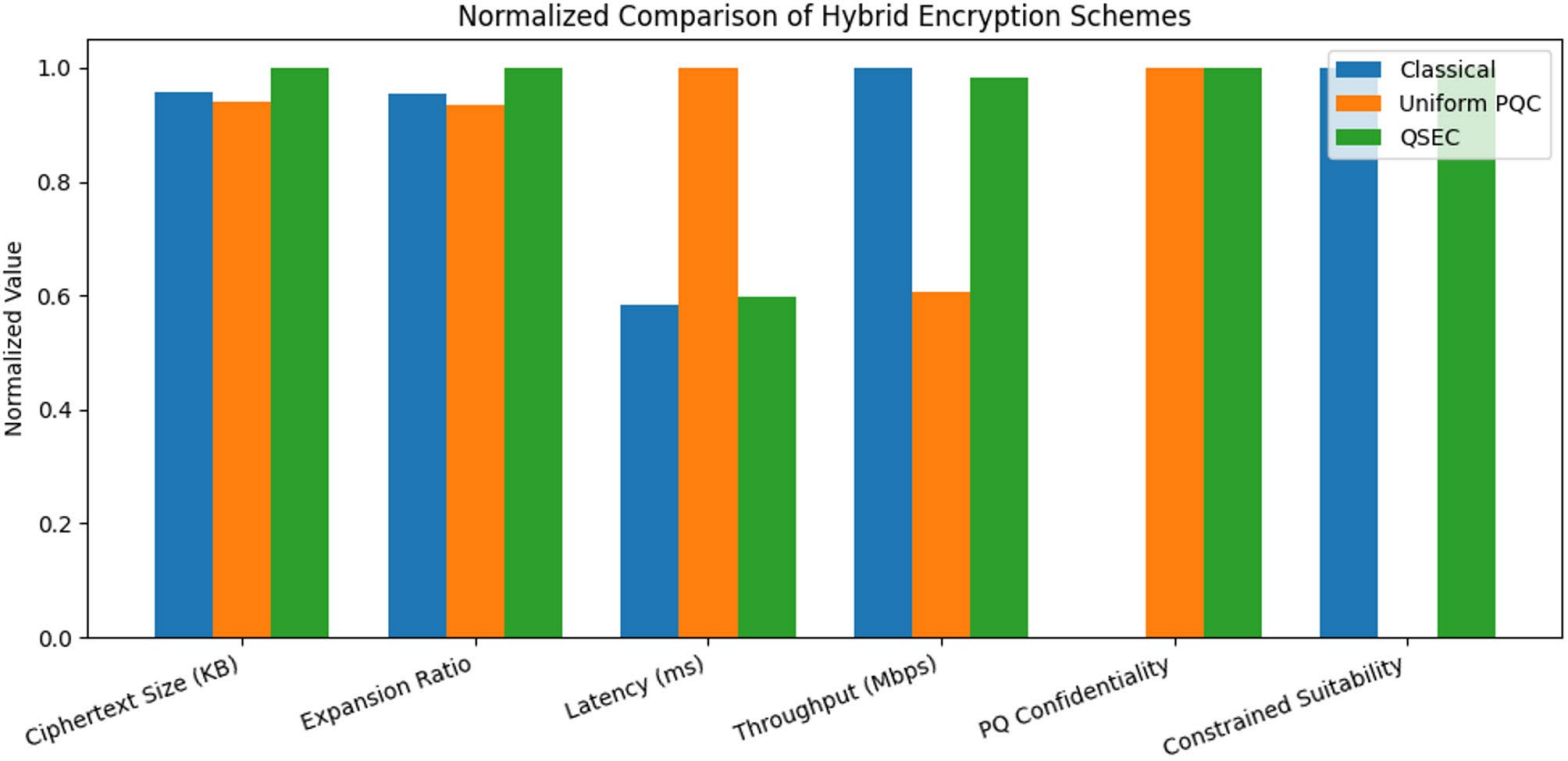
Normalized comparison of hybrid encryption schemes.

Figure 5 provides a normalized performance comparison between Classical encryption, Uniform PQC implementation, and the proposed QSEC hybrid scheme in terms of ciphertext size, expansion ratio, latency, throughput, post-quantum confidentiality, and suitability to constrained-environment implementation. Compared to the Uniform PQC model, the Uniform PQC model has a higher latency and lower throughput, indicating a higher computational overhead, and thus gives the highest post-quantum confidentiality. The classical approach is characterized by high throughput and reduced latency and has no quantum confidentiality. Conversely, the suggested QSEC hybrid scheme is not only similar to Classical performance on throughput, but also has a low latency and better ciphertext efficiency; at the same time, it can support total post-quantum confidentiality. Also, it indicates to be more suitable for limited environments than Uniform PQC. All in all, the number indicates that the suggested hybrid QSEC method provides a good compromise between quantum security and the efficiency level of the operation.

**Table 12:** Threat mitigation effectiveness and residual risk assessment.

Threat class	Dominant mitigating module	Attack capability (0–10)	Expected impact (0–10)	Mitigation effectiveness (%)	Residual risk score
Passive eavesdropping	**Module 2: QSEC**	3.2	7.5	**99.85**	**0.011**
Active MITM	**Module 2: QSEC**	6.8	8.4	**99.12**	**0.074**
Quantum cryptanalytic attack	**Module 2: QSEC**	9.5	9.8	**99.92**	**0.008**
Encrypted analytics inference	**Module 3: HM-Intel**	6.0	8.7	**99.78**	**0.019**
Replay attack	**Module 1: DDP-QKA**	5.4	6.9	**98.96**	**0.072**
Insider compromise (single node)	**Module 4: QFTG**	7.1	8.1	**98.91**	**0.088**
Insider coalition (≥ 3 nodes)	**Module 4: QFTG**	8.6	9.0	**97.84**	**0.195**
Post-event forensic attack	**Module 4: QFTG**	8.2	9.1	**99.89**	**0.010**
DoS / jamming	**Module 1: DDP-QKA**	7.9	7.8	**95.34**	**0.364**

Significant values are in bold.

Table 12 provides the detailed analysis of the key threat categories, their score of attack ability, the impact expectation (010 scale), the most prevalent mitigating module, mitigating performance, and the resulting score of the remaining risk. The primary defense against passive eavesdropping, active MITM, and quantum cryptanalytic attacks is Module 2: QSEC, with very high effectiveness levels of above 99, and very low residual risks (0.011, 0.074, and 0.008, respectively), which shows significantly strong resistance to quantum adversaries with high capabilities. Module 3: HM-Intel helps to prevent encrypted analytics inference with 99.78 success and 0.019 low residual risk, which shows privacy-sensitive intelligence processing. Module 1: DDP-QKA primarily mitigates replay attacks (high mitigation, 98.96, and low residual risk, 0.072), and DoS/jamming (high mitigation, 95.34, and relatively high residual risk, 0.364), but is disruptive at the network layer. Module 4: QFTG handles insider threats, with single-node compromise resulting in a residual risk at 0.088 and a mitigation of 98.91, and insider coalition ( > = 3 nodes) providing a higher level of systemic risk, at 97.84 and 0.195, respectively. The probability of post-event forensic attacks is highly discouraged (99.89) with insignificant exposure (0.010). In general, the findings indicate that the built-in multi-module architecture can greatly decrease operational risk on a wide variety of attack vectors, with only distributed insider and large-scale availability attacks exhibiting relatively high but manageable residual risk levels.

**Fig. 6:**
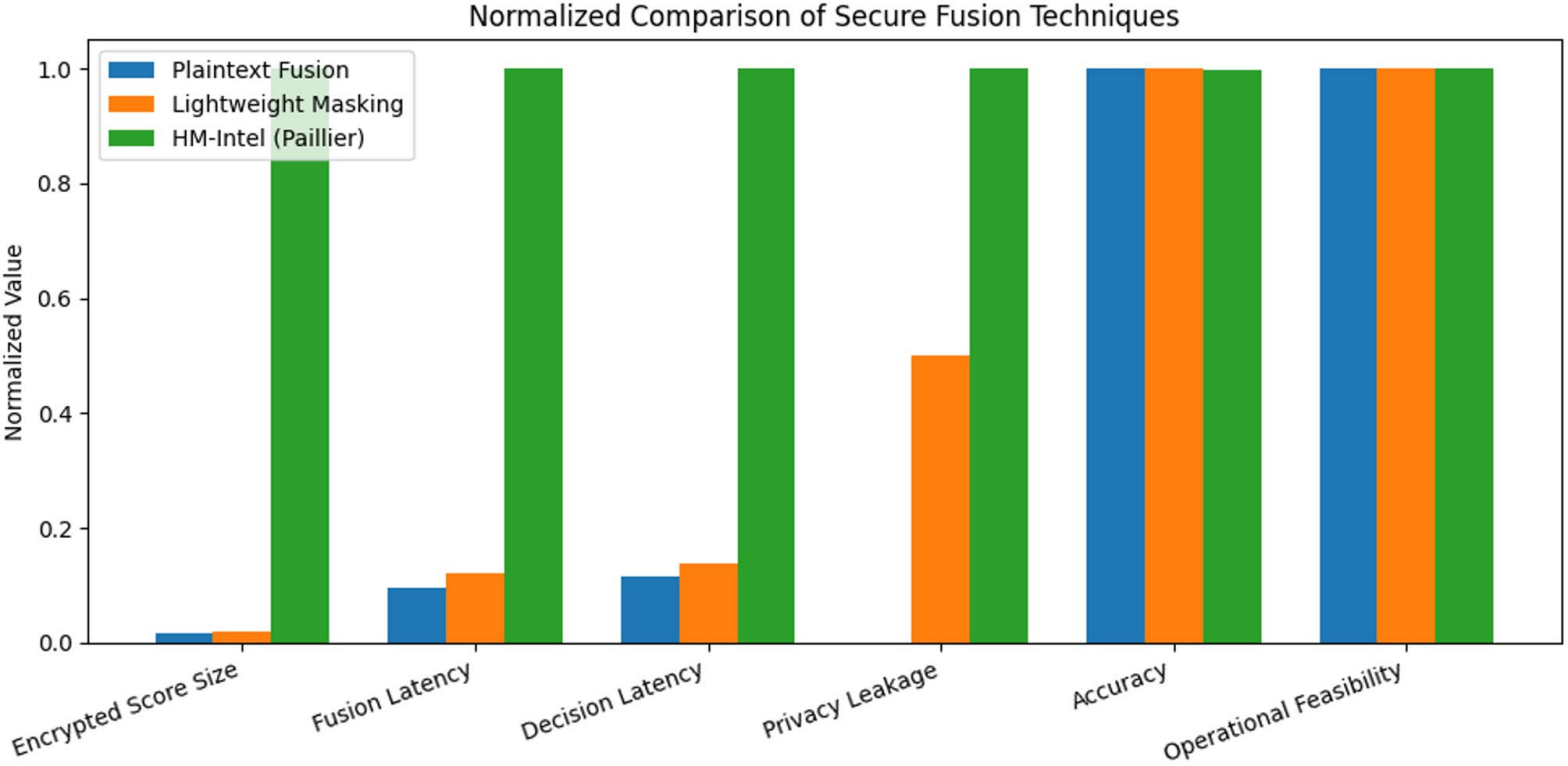
Normalized comparison of secure fusion techniques.

The normalized comparison of Plaintext Fusion, Lightweight Masking, and the proposed HM-Intel (Paillier-based) secure fusion technique is as in Fig. 6 in terms of encrypted score size, fusion latency, decision latency, privacy leakage, accuracy, and operational feasibility. Plaintext fusion has low latency and score size, but no protection against privacy, which leads to a maximum amount of privacy leakage. Lightweight masking lowers the exposure; still, it exhibits moderate leakage and small robustness. Contrary to this, the HM- Intel scheme has a privacy leakage of almost zero and has full operational feasibility as well as accuracy. Even though it comes at a relatively increased fusion and decision latency since it uses homomorphic processing, the security benefits easily override the performance overhead. On the whole, the figure indicates that the suggested HM-Intel approach offers the most privacy-sensitive intelligence fusion with reasonable computational cost, which is indeed viable in environments with secure defense-level analytics. Overall, the findings confirm that the DEF-CRYPT-Q architecture strikes a proper balance between security, latency, and communication performance, especially when applied to mission-critical defense communications.

## Conclusion

The paper presented a new quantum-resilient and privacy-safe cryptographic system, DEF-CRYPT-Q, which is built to provide security to distributed, AI-based, and multi-domain defense communication systems against the emerging classical and quantum threats. The proposed architecture incorporates context-aware lightweight encryption, lattice-based post-quantum key establishment and authentication, homomorphic encrypted intelligence processing, and federated trust governance, which is appropriate to balance the security strength and operational efficiency of a heterogeneous battlefield with limited resources. Both experimental and statistical tests demonstrates that the adaptive design minimizes encryption latency, key-management overhead, bandwidth growth, and energy usage without compromising on high quantum resistance, improved forward secrecy, and high insider threat containment. DEF-CRYPT-Q has a better performance, scalability, and adversarial resilience trade-off than the traditional AES-RSA systems and uniform post-quantum deployments. As a whole, the framework offers a future-ready and scalable cryptographic template that is in line with the strategic defense modernization objectives to support high mobility and mission-critical infrastructures of secure communications in the quantum age.

The proposed DEF-CRYPT-Q algorithm shows better performance in terms of security, latency, and energy consumption during simulated communication scenarios for defense purposes. However, the experiment outcomes have been obtained in a simulation environment without any formal proof regarding security and without deploying the algorithm to actual communication networks. Thus, the outcomes should be analyzed keeping in mind the limitations of the experimental environment.

## Data Availability

The datasets used during the current study are available from the corresponding author on reasonable request.
